# Editorial: Risk of COVID-19 Transmission to Oral Healthcare Providers and Their Patients

**DOI:** 10.3389/froh.2022.894530

**Published:** 2022-04-13

**Authors:** Marilynn L. Rothen, Joana Cunha-Cruz

**Affiliations:** ^1^Department of Oral Health Sciences, School of Dentistry, University of Washington, Seattle, WA, United States; ^2^Department of Clinical and Community Sciences, School of Dentistry, University of Alabama at Birmingham, Birmingham, AL, United States

**Keywords:** COVID-19, infection control, pandemic (COVID-19), oral health, dental care, qualitative study, quantitative study, COVID-19 guidelines

A little <2 years ago, at the start of the global coronavirus disease 2019 (COVID-19) pandemic, researchers began to investigate the impact of the severe acute respiratory syndrome coronavirus 2 (SARS-CoV-2) on the oral healthcare workforce and the patients they serve. There was concern that the infectious nature of the virus if spread though respiratory and oral secretions, combined with the use of aerosolizing procedures in dentistry, could put providers and patients in the dental environment at high risk of contracting and spreading the viral infection. In many areas, as governments limited dental practices to only emergency care for many weeks, the ramifications of postponed care on patients and the economic cost to practices added additional risks and stressors meriting evaluation.

Just prior to the start of the pandemic, Otieno et al. explored infection control practices among private practice dentists in Nairobi providing a window into the preparedness of oral healthcare providers to face the risks presented by SARS-CoV-2. They suggest, based on their findings, that closing dental facilities at the start of the pandemic may have been a necessary pause to allow dental offices to develop and adopt strategies for safer practices in the face of the unknown transmission risk of SARS-CoV-2 in the dental office. As the pandemic began to increase its global reach, dental practitioners started to routinely adopt additional precautions as advised by national and international health organizations. Pittayapat et al. illustrate a proposed model for risk assessment and risk reduction in a Thai hospital. Luo et al. describe a protocol to decrease cross-infection in dental clinics to help ameliorate the outbreak of COVID-19 and recommend adoption of these clinical guidelines in China.

The uncertainty created by the outbreak of the COVID-19 pandemic was explored by Humphris et al. 3 months into the pandemic. Their survey of both primary dental care staff and dental trainees sheds light on the pandemic's effect on provider health and wellbeing, including preparedness for coping with uncertainty and financial insecurity. They propose a theoretical model for understanding the pathways to depressive symptomatology and possible burnout in such periods of high uncertainty. Supplementing their quantitative findings, this same group published a qualitative examination authored by Knights et al. of the lived experience of dental teams during the pandemic. Dental trainees experienced professional isolation and great uncertainty for their professional future. The uncertainty and confusion, both medium and long term, brought on by the pandemic highlights the need for measures to support the wellbeing of oral healthcare professionals.

Taken together, these reports help us to understand the impact of fear, uncertainty, and economic hardship on dental care providers during a global pandemic. The work of Pittayapat et al. and Luo et al. contribute to the body of evidence-based guidelines to reduce the risk of transmission to patients and providers during clinical care. While clinical guidelines help to address providers' and patients' fear for their physical health and safety, they do not address the mental health toll as discussed by Humphris et al. and Knights et al. Dissemination of best practices for times of uncertainty due to a pandemic might include dental trainee preparation for unanticipated global emergencies that addresses mental health and wellbeing in addition to physical wellbeing. Future research can focus on quantifying the risk of transmission to patients and providers during dental treatment, unanticipated hazards from extended wearing of extensive personal protective equipment (PPE) to prevent or limit exposure to droplets and aerosols, measures to support mental health and wellbeing during global emergencies and prevent burn out that may contribute to workforce shortages. Research Topic of peer reviewed articles in volume one of the Research Topic: *Risk of COVID-19 Transmission to Oral Healthcare Providers and their Patients* sets the stage for volume two to focus on the many lessons learned about the provision of oral healthcare during a global pandemic.

## Author Contributions

Both authors wrote the manuscript and approved the final version of the manuscript.

## Conflict of Interest

The authors declare that the research was conducted in the absence of any commercial or financial relationships that could be construed as a potential conflict of interest.

## Publisher's Note

All claims expressed in this article are solely those of the authors and do not necessarily represent those of their affiliated organizations, or those of the publisher, the editors and the reviewers. Any product that may be evaluated in this article, or claim that may be made by its manufacturer, is not guaranteed or endorsed by the publisher.

